# Distribution of Glyphosate-Resistance in *Echinochloa crus-galli* Across Agriculture Areas in the Iberian Peninsula

**DOI:** 10.3389/fpls.2021.617040

**Published:** 2021-02-12

**Authors:** José G. Vázquez-García, Antonia M. Rojano-Delgado, Ricardo Alcántara-de la Cruz, Joel Torra, Ignacio Dellaferrera, João Portugal, Rafael De Prado

**Affiliations:** ^1^Department of Agricultural Chemistry and Edaphology, University of Córdoba, Córdoba, Spain; ^2^Departamento de Química, Universidade Federal de São Carlos, São Carlos, Brazil; ^3^Department d’Hortofruticultura, Botànica i Jardineria, Agrotecnio, Universitat de Lleida, Lleida, Spain; ^4^Faculty of Agricultural Sciences, National University of the Litoral, Esperanza, Argentina; ^5^National Scientific and Technical Research Council, Godoy Cruz, Argentina; ^6^Biosciences Department, Polytechnic Institute of Beja, Beja, Portugal; ^7^VALORIZA-Research Centre for Endogenous Resource Valorization, Polytechnic Institute of Portalegre, Portalegre, Portugal

**Keywords:** barnyard grass, enhanced metabolism, glyphosate, non-target-site resistance (NTSR), resistance mechanisms, target-site resistance (TSR)

## Abstract

The levels of resistance to glyphosate of 13 barnyard grass (*Echinochloa crus-galli*) populations harvested across different agriculture areas in the Southern Iberian Peninsula were determined in greenhouse and laboratory experiments. Shikimate accumulation fast screening separated the populations regarding resistance to glyphosate: susceptible (S) E2, E3, E4, and E6 and resistant (R) E1, E5, E7, E8, E9, E10, E11, E12, and E13. However, resistance factor (GR_50_ E1–E13/GR_50_ E6) values separated these populations into three groups: (S) E2, E3, E4, and E6, (R) E1, E5, E7, E8, and E9, and very resistant (VR) E10, E11, E12, and E13. ^14^C-glyphosate assays performed on two S populations (E2 and E6) showed greater absorption and translocation than those found for R (E7 and E9) and VR (E10 and E12) populations. No previous population metabolized glyphosate to amino methyl phosphonic acid (AMPA) and glyoxylate, except for the E10 population that metabolized 51% to non-toxic products. The VR populations showed two times more 5-enolpyruvylshikimate-3-phosphate synthase (EPSPS) activity without herbicide than the rest, while the inhibition of the EPSPS activity by 50% (I_50_) required much higher glyphosate in R and VR populations than in S populations. These results indicated that different target-site and non-target-site resistance mechanisms were implicated in the resistance to glyphosate in *E. crus-galli*. Our results conclude that resistance is independent of climate, type of crop, and geographic region and that the level of glyphosate resistance was mainly due to the selection pressure made by the herbicide on the different populations of *E. crus-galli* studied.

## Introduction

Weeds are the main constraint in global food production and have a pivotal role in reducing quality and yield in the most important crops worldwide (Oerke, 2006). Weed control strategies have been constantly changing over recent decades through cropped areas with a tendency to monoculture without herbicide rotation, such as perennial crops, or large irrigated and horticultural crops. This scenario has provoked a decrease in herbicide efficacies due to the evolution of weed resistant biotypes. In particular, there was a quick shift in cases of weed species resistant to the single 5-enolpyruvylshikimate-3-phosphate synthase (EPSPS) inhibiting herbicide glyphosate (group 9, HRAC and WSSA), currently the most extensively herbicide used over the world (Baylis, 2000). Since the evolution of a glyphosate resistant (GR) weed was reported for the first time (Pratley et al., 1996), 51 weed species were documented to have populations with evolved herbicide resistance over millions of hectares of the best crop producing areas around the globe (Heap, 2020). Glyphosate has been widely used in GR crops in many American countries, while this herbicide is used especially in the European Mediterranean in perennial crops, corn, and rice in direct sowing and large horticultural crops, among others (Antier et al., 2020). It is well-known that glyphosate is a non-selective herbicide that it is absorbed through leaves. The enzyme EPSPS (EC 2.5.1.19) is the target-site of glyphosate in plants. This enzyme catalyzes, in the shikimic acid pathway, the conversion of phosphoenolpyruvate and shikimate-3-phosphate into inorganic phosphate and 5-enolpyruvylshikimate-3-phosphate. Its inhibition prevents the biosynthesis of phenylalanine, tyrosine, and tryptophan, aromatic amino acids (Franz et al., 1997). The resistance mechanisms are broadly divided into non-target-site resistance (NTSR) and target-site resistance (TSR) (Gaines et al., 2020). TSR implies conformational changes in the target-proteins of herbicides that result from deletion or amino acid substitution, but also gene overexpression or amplification that increases target protein abundances (Gaines et al., 2020). NTSR covers those mechanisms not related to the enzymes targeted by herbicides. Often, NTSR mechanisms act reducing to a sublethal dose the herbicide that reaches a target protein and may involve reduced absorption/translocation of the herbicide, vacuolar sequestration, or enhanced metabolism (metabolic herbicide resistance) (Ghanizadeh and Harrington, 2017).

The Iberian Peninsula, with more than 5,000,000 ha, followed by Italy (2,500,000 ha), was the most important member state of the EU-28 Mediterranean Region in terms of perennial, corn, and rice crops in direct sowing and large horticultural crops in 2017 (Antier et al., 2020). The common climate, absence of crop rotation, and few herbicides being widely used resulted in their fields having similar glyphosate resistant weeds. Currently, *Conyza bonariensis*, *Conyza canadensis*, *Conyza sumatrensis*, *Hordeum murinum, Lolium multiflorum*, *Lolium perenne*, *Lolium rigidum*, and *Sorghum halepense* have evolved resistance to glyphosate in Iberian Peninsula (Heap, 2020). Nevertheless, since 2018, farmers have been complaining about the appearance of a new glyphosate resistant grass species, identified as *Echinocloa crus-galli*.

*Echinochloa crus-galli* (L.) P. Beauv is an annual C4 grass weed reported as a hexaploid species, whose karyotype is 2*n* = 6*x* = 54 chromosomes (Ye et al., 2020). The plant has dull green leaves often with conspicuous anthocyanin pigmentation, glabrous compressed sheaths, with no ligules and auricles; they form a clump with prostrate tillers reaching up to 150 cm in height and reproduces by caryopses disposed in erected panicles (Maun and Barrett, 1986; Damalas et al., 2008). Fertilization occurs mainly by self-pollination; however, a certain degree of crossbreeding can occur, facilitated by wind. High levels of homozygosity within populations result from self-fertilization together with a relatively low degree of heterozygosity in polymorphic loci (Maun and Barrett, 1986). It has a high tillering capacity, being also a very prolific species (Owen et al., 2020); these characteristics, added to the fact that seeds can easily disperse, are dormant, and it can flower under a wide photoperiod range, make it a very successful weed (Maun and Barrett, 1986). This species has biological and ecological similarities with rice and for this reason is one of the main rice weeds all over the world (Tian et al., 2020), but in the Iberian Peninsula it also acts as weed in soybean, maize, and other crops (Dorado et al., 2009). This is a particular concern because it is among the top 15 weed species with herbicide resistance worldwide (Yang et al., 2017) with cases reported in 23 countries, principally in rice but also in other crops, such as corn, orchards, and perennial crops. Among the herbicidal modes of action to which *Eleusine indica* has been reported as being resistant are the inhibitors of the acetolactate synthase, acetyl-CoA carboxylase, 1-deoxy-D-xyulose 5-phosphate synthase, EPSPS, photosystem II, cellulose, lipids, microtubules, a very long chain fatty acid, as well as synthetic auxins (Heap, 2020).

This study determined whether *E. crus-galli* populations, infesting several perennial and annual crops in the Iberian Peninsula, are resistant to glyphosate, as well as the resistance mechanisms present, particularly NTSR mechanisms (absorption, translocation, and metabolism). EPSPS enzyme activity data were used to infer putative TSR mechanisms present in the studied populations.

## Materials and Methods

### Plant Material

Mature seeds of *E. crus-galli* were collected between 2018 and 2019 in perennial crop fields (olive, citrus, vineyards, and pomegranates-tree) and annual crops (rice and corn) from the south of the Iberian Peninsula (Table 1), where farmers reported control failures of this species with glyphosate after more than 10 years of application. Thirteen populations were collected and taxonomically characterized, and we obtained historical records of field application only for some populations due to a lack of good record keeping in other cases. The seeds of each population were harvested from at least 25 adult plants in georeferenced 50 m^2^ areas (Table 1). Seeds were cleaned and stored at 4°C for further testing. Germination of the different populations was very irregular and was between 40 and 80%.

**Table 1:** Distribution of *Echinochloa crus-galli* across agriculture areas in the Southern Iberian Peninsula and its main management characteristics with glyphosate.

Pop.	Crop	Country	GPS coordinates	Year application^a^/dose^b^	Year harvested
E1	Olive grove	Spain	37°40'32.5''N 4°14'23.0''W	10/720	2018
E2	Citrus orchard	Spain	37°42'06.6''N 5°18'48.7''W	Organic	2019
E3	Olive grove	Spain	37°31'05.7''N 4°50'30.9''W	5/540	2018
E4	Olive grove	Spain	37°42'30.3''N 4°30'45.3''W	3/540	2018
E5	Orchard	Spain	37°38'06.9''N 4°21'54.1''W	15/540–720	2019
E6	Runnel (non crop)	Portugal	38°01'12.4''N 7°46'08.0''W	No	2019
E7	Citrus orchard	Spain	37°45'24.2''N 5°15'56.9''W	12/1080	2019
E8	Citrus orchard	Spain	37°41'57.7''N 5°18'28.7''W	10/720	2019
E9	Rice	Spain	36°22'16.0''N 5°52'40.6''W	12/1080	2019
E10	Pomegranates-tree	Portugal	38°06'02.4''N 7°49' 21.9''W	15/1080	2019
E11	Corn	Spain	36°19'32.1''N 5°47'34.5''W	12/720-1080	2019
E12	Corn	Portugal	37°54'54.5''N 8°21'47.8''W	15/1080	2019
E13	Vineyard	Portugal	37°54'13.2''N 7°58'23.8''W	12/720-1080	2019

a Usually farmers applied two times year^−1^ in perennial crops, in the last time (5 years) in autumn, herbicides such as flazasulfuron [acetolactate synthase (ALS) inhibitor] and oxyfluorfen [protoporphyrinogen oxidase (PPO) inhibitor] plus glyphosate are applied. In spring, commonly MCPA plus glyphosate are applied. On the other hand, in annual crops such as rice and corn, the glyphosate is applied only one time cicle^−1^ in pre-sowing.

b g ae ha^−1^.

The climate in central Andalusia (Southern Spain) and Alentejo (Center of Portugal) typically has long, hot, and arid summers and winters that are short, cold, and partly cloudy. Throughout the year, the temperature generally varies from 6 to 35°C and rarely drops below 2°C or rises above 45°C. All fields where seeds were collected were irrigated with river or swamp water that ranges between 1,500 and 6,000 L ha^−1^. The types of soils were highly variable between sandy and clay.

Fifteen-cm-diameter petri dishes were conditioned with two layers of moistened (5 ml distilled water) filter paper to germinate the seeds of the *E. crus-galli* populations. Petri dishes were kept in a germination chamber calibrated at 28/18°C (day/night), 16-h photoperiod, 850 μmol m^−2^ s^−1^ light intensity, and 80% relative humidity. Once germinated, seedlings were transplanted individually in 250 ml punnet pots (peat/sand, 2:1 v/v) and taken to a greenhouse maintaining the same temperature and photoperiod regime as in the germination chamber (Fernández-Moreno et al., 2017a).

### Shikimate Accumulation Fast Screening

Five samples (50 mg of 4 mm diameter leaf discs) of each *E. crus-gaalli* population were taken from a pool of young and fully expanded leaves from at least 10 plants (Vázquez-García et al., 2020a). Leaf discs of each sample were saved in 2 ml tubes containing 1 ml of different glyphosate concentrations (0 and 1000 μM) prepared in 1 mM ammonium dihydrogenphosphate (pH 4.4). Sample tubes were incubated at 25°C and light intensity of 850 μmol m^−2^ s^−1^. Shikimic acid was extracted following the methodology of Shaner et al. (2005). Accumulation was estimated from the difference between the shikimic acid concentration in treated and untreated plants based on a calibration curve with known concentrations of standard shikimic acid (Sigma-Aldrich Co., Saint Louis, MO, United States). Two technical replicates were analyzed from each sample and the results were expressed in μg g^−1^ fresh weight.

### Dose-Response Assays

Plants at the three to four leaf stages of the *E. crus-galli* populations were treated with nine glyphosate (Roundup Energy, 450 g ae L^−1^ as isopropylamine salt) doses ranging from 0 to 3,000 g ae ha^−1^. Herbicide applications were done in a herbicide treatment cabinet with output volume of 200 L ha^−1^ at a pressure of 250 kPa. Moving-boom of the cabinet has a Teejet 8002-EVS nozzle positioned 50 cm above the plant canopy. Sets of 10 plants of each population were treated for each dose of herbicide, and the experiments were repeated twice. Herbicide response (weight reduction and mortality) were determined 21 days after the treatments (DAT) and transformed in percentage with respect to the controls (Vazquez-Garcia et al., 2020b).

### ^14^C-Glyphosate Uptake and Translocation

The second or third leaf of eight plants (five and three for quantitative and qualitative analyzes, respectively) of the E2, E6, E7, E9, E10, and E12 populations was covered with aluminum envelopes. Plants were sprayed with 360 g ae ha^−1^ of formulated glyphosate (cold treatment) and 30 min later, once herbicide solution dried, the aluminum was removed. After, 1-μl drop of ^14^C-glyphosate (glycine-2-^14^C, 95% radiochemical purity, 273.8 MBq mmol^−1^specific activity, Institute of Isotopes Co., Ltd., Budapest, Hungary) + formulated glyphosate (hot treatment) per plant was deposited on the adaxial surface of these leaves using a micro syringe (Hamilton PB6000 Dispenser). The hot solution had 100,000 dpm μl^−1^ specific activity and 360 g ae ha^−1^. Four DAT, the non-uptake ^14^C-glyphosate was washed three times with water: acetone (1:1 v/v; 1 ml each time). Wash solutions were recovered in ml scintillation vials and 2 ml of scintillation cocktail was added.

Plants were removed from the punnet pots and impurities in the roots were carefully washed with distilled water. Quantitative analysis plants were sectioned into treated leaf, rest of the aerial part of the plant, and roots. Plant sections were saved in filter paper cones, dried at 60°C during 4 days and burned individually in an automatic oxidizer (Packard Tri Carb 307, Packard Instruments, Meriden, United States) during 3 min. The ^14^CO_2_ released during combustion was captured in 18 ml of radioactive dioxide absorber solution (Carbosorb-E®, Perkin-Elmer) and liquid scintillation cocktail (Permafluor®, Perkin-Elmer; 1:1, v/v). Radioactivity of wash solutions and combustion was quantified by liquid scintillation spectrometry (10 min). Experiments had a randomized design and the absorption and translocation percentages were calculated according to Alcántara-de la Cruz et al. (2021).

The three plants of each population reserved for the qualitative analysis of ^14^C-glyphosate translocation were fixed on filter paper sheets (12.5 cm × 25 cm), pressed and dried at room temperature for 1 week. The dried plants were then exposed to a phosphor storage screen for 13 h in the dark. Radioactivity distribution within plants was scanned in a storage phosphor system (Cyclone Plus, Perkin-Elmer).

### Glyphosate Metabolism

Ten plants at the four-leaf stage of the E2, E6, E7, E9, E10, and E12 populations were sprayed with glyphosate at 360 g ae ha^−1^. Other groups of plants (the same number of plants) were sprayed only with water to be used as control. Four DAT, whole plants were removed from the punnet pots, carefully washed with distilled water, packed in aluminum foil envelopes, and immediately frozen in liquid N_2_. The samples were stored at 40°C until processing for analysis. The extraction of amino methyl phosphonic acid (AMPA), formaldehyde, glyphosate, glyoxylate, and sarcosine as well as its quantification by reversed polarity capillary electrophoresis were performed according to Rojano-Delgado et al. (2010). The concentrations of each compound were determined using calibration curves with known concentrations of standard compounds (Sigma-Aldrich, Madrid, Spain). Data were expressed as percentages of the sum of glyphosate plus metabolites recovered.

### EPSPS Enzyme Activity Assays

The EPSPS activity was assayed in the E6, E7, E9, E10, and E12 populations. Leaf tissue samples were taken from four leaf stage plants up to complete 5 g per population. Samples were stored at 40°C until analyses, when they were macerated in a mortar until obtaining fine powder. The extraction of the target enzyme of the glyphosate, as well as the determination of the total soluble protein (TPS, basal activity without glyphosate) and the EPSPS inhibition rate by adding increased concentrations of glyphosate (0, 0.1, 1, 10, 100, and 1000 μM) were performed following the detailed methodology by Dayan et al. (2015). For each glyphosate concentration, three technical replicates of each population were assayed. Experiment was repeated twice and the results were given as a percentage relative to the control (0 μM glyphosate) of the amount (μmol) of inorganic phosphate (Pi) released per μg of TSP min^−1^ (μmol Pi μg^−1^ TSP min^−1^).

### Statistical Analyses

The three-parameter regression function, *y* = *d*/{1+exp.[*b*(log *x* – log *e*)]}, was used to estimate the weight reduction, plant mortality, and EPSPS inhibition at a rate of 50% (GR_50_, LD_50_, and I_50_, respectively), by fitting their respective percentage data in the “drc” package of the R software environment (Keshtkar et al., 2021). The function parameters represent: “*b*” is the relative slope of the curve, “*d*” is the upper limit of “*y*,” “*e*” is the herbicide rate that reduces “*y*” by 50%, and “*y*” is the dry weight, plant survival, or EPSPS inhibition of a given population. Resistance levels (RF) were calculated for each variant of “*y*” as the ratio between the “*e*” of the resistant populations to the “*e*” of the representative susceptible population.

For the rest of the data, the normal error distribution and the homogeneity of the variance were verified for each set. Then ANOVAs were performed and when the value of *p* was <0.05, the means were separated by the Tukey’s test.

## Results

### Shikimate Accumulation Fast Screening

**Figure 1:**
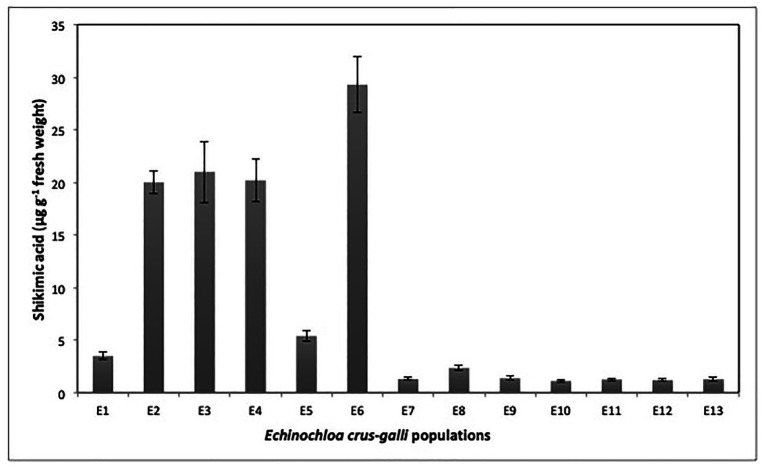
Shikimate accumulation values in 13 *Echinochloa crus-galli* populations treated with glyphosate at 1000 μM.

The accumulation of shikimic acid differed between *E. crus-galli* populations. The populations E2, E3, E4, and E6 accumulated high rates of shikimic acid. The highest accumulation (29.3 μg shikimic g^−1^) was recorded at 1,000 μM glyphosate in the E6 population. Regarding populations resistant to glyphosate, we observed two groups; the first was formed by populations E1, E5, E7, E8, and E9, which accumulated low rates of shikimic acid that varied between 1.4 and 5.4 μg g^−1^ fresh weight. The second group was made up of populations E10, E11, E12, and E13 that accumulated very low rates of shikimate, ranging from 1.1 to 1.3 μg shikimic acid g^−1^ fresh weight (Figure 1).

### Dose Response Assays

**Figure 2:**
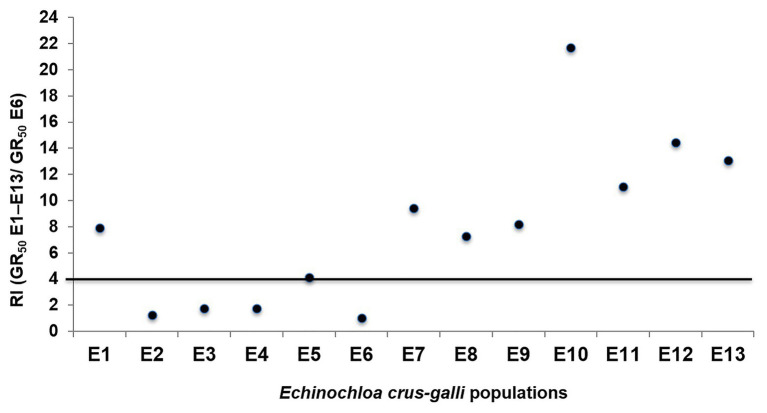
Representation of the resistance factor (RF; GR_50_ E1–E13/GR_50_ E6) values of different *Echinochloa crus-galli* populations. Populations above the line were considered glyphosate-resistant.

The 13 *E. crus-galli* populations were grouped in: glyphosate-susceptible (S), −resistant (R), and -very resistant (VR), considering their GR_50_. The group of S populations (E2, E3, E4, and E6) had RF values less than 4 and the LD_50_ values were also very low and less than the field label dose (1.08 kg ae ha^−1^). However, the nine resistant populations survived the field doses and their LD_50_ ranged from 1532 (E1) to 2892 (E10) g ae ha^−1^. The GR_50_ values separated the resistance level into two groups; first group formed by R populations E1, E5, E7, E8, and E9 with RF values between 6.9 and 9.4 and a second group of VR populations E10, E11, E12, and E13 with RF values between 11 (E11) and 21.7 (E10) (Table 2; Figure 2).

**Table 2:** Parameters^a^ of the equations used to calculate the glyphosate rates (g ae ha^−1^) required for a 50% reduction in dry weight (GR_50_) or survival plants (LD_50_) of 13 *Echinochloa crus-galli* populations.

Pop.	b	d	GR_50_^b^	RF^c^	b	d	LD_50_^b^	RF^c^
E1	1.4	91.7	317.6 ± 53.2	7.9	21.4	100.0	1532.9 ± 41.5	12.3
E2	1.6	100.7	50.9 ± 5.8	1.3	14.9	100.5	157.6 ± 11.2	1.3
E3	1.7	104.3	71.0 ± 7.2	1.8	39.3	100.0	363.8 ± 32.0	2.9
E4	1.9	99.1	69.7 ± 7.4	1.7	46.3	100.0	296.0 ± 3.6	2.4
E5	0.9	97.9	166.3 ± 32.8	6.7	36.7	100.0	1528.6 ± 66.2	12.2
E6	3.1	100.1	40.3 ± 3.0	-----	11.8	100.1	125.0 ± 4.6	----
E7	1.4	99.7	379.1 ± 47.3	9.4	26.3	100.0	2447.3 ± 10.4	19.6
E8	1.7	99.7	293.3 ± 33.1	7.3	28.9	100.0	2000.0 ± 36.5	16.0
E9	1.1	98.3	328.6 ± 55.8	8.2	43.2	100.0	2465.0 ± 20.5	19.7
E10	3.6	94.5	873.2 ± 43.6	21.7	30.2	100.0	2893.0 ± 11.4	23.1
E11	0.9	100.2	444.8 ± 8.5	11.0	74.4	100.0	2432.5 ± 12.0	19.5
E12	1.3	97.2	581.2 ± 7.3	14.4	22.9	100.0	2098.2 ± 33.7	16.8
E13	1.4	100.6	525.2 ± 11.5	13.0	22.9	99.9	2098.2 ± 36.9	16.8

a *y* = *d*/{1+exp[*b*(log x – log *e*)]}, where *b* is the relative slope of the curve, *d* is the upper limit of “*y*,” *e* is the herbicide rate that reduces “*y*” by 50% and “*y*” is the dry weight (GR_50_) or plant survival (LD_50_) of a given population.

b Mean ± SEM.

c RF = Resistance factor (R/S−E6) calculated using the GR_50_ or LD_50_ values.

### ^14^C-Glyphosate Uptake, Translocation, and Visualization

**Figure 3:**
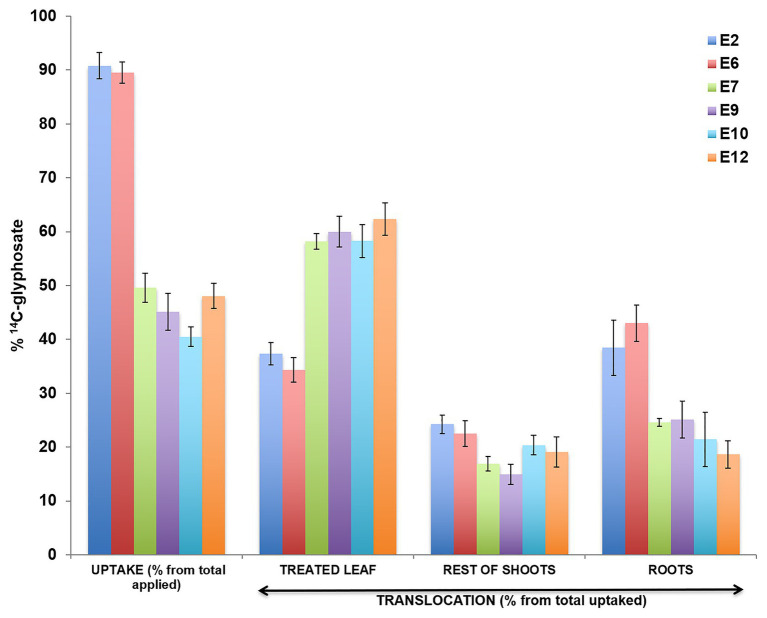
Absorption and translocation of ^14^C-glyphosate (%) at 96 h after treatment in different *Echinochloa crus-galli* populations, glyphosate-resistant (R; E7 and E9), -very resistant (VR; E10 and E12), and -susceptible (S; E2 and E6).

**Figure 4:**
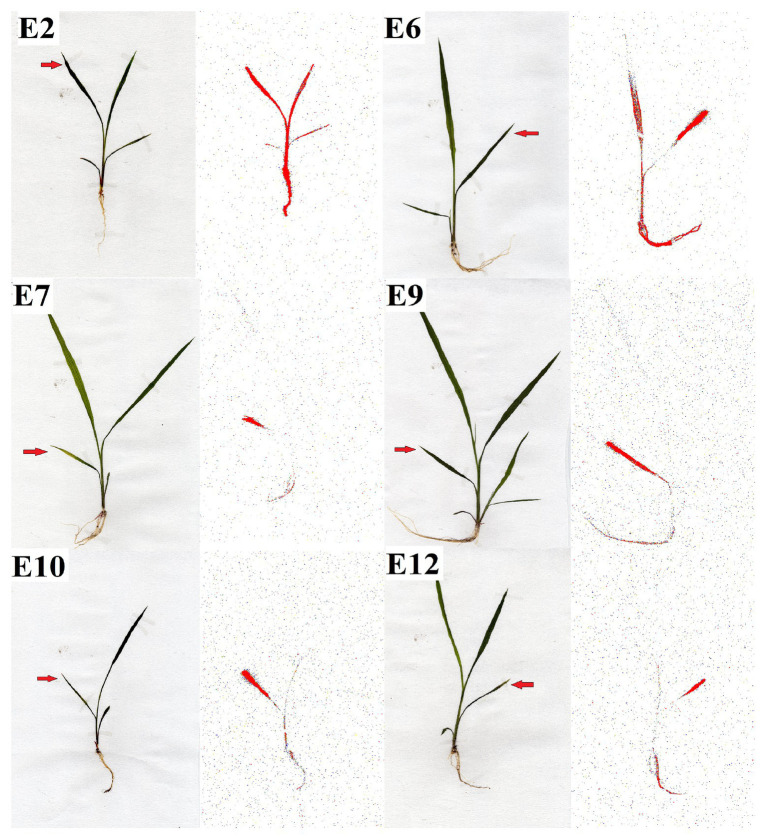
Visualization of ^14^C-glyphosate in resistant (R; E7 and E9), -very resistant (VR; E10 and E12), and -susceptible (S; E2 and E6) *Echinochloa crus-galli* populations 96 h after an application to the treated leaf.

The ^14^C-glyphosate recovered in two S (E2 and E6), two R (E7 and E9), and two VR (E10 and E12) populations ~90–96% after 4 DAT. The uptake rate of ^14^C-glyphosate was higher in the S populations E2 and E6 compared with the resistant populations. In addition, the S populations moved more ^14^C-herbicide from the treated leaf to the rest of the shoots (rest of the aerial part of the plant plus root system) was shown in compared with the R and VR populations (Figure 3). ^14^C-glyphosate visualization (red color) confirmed previous results (Figure 4).

### Glyphosate Metabolism

**Figure 5:**
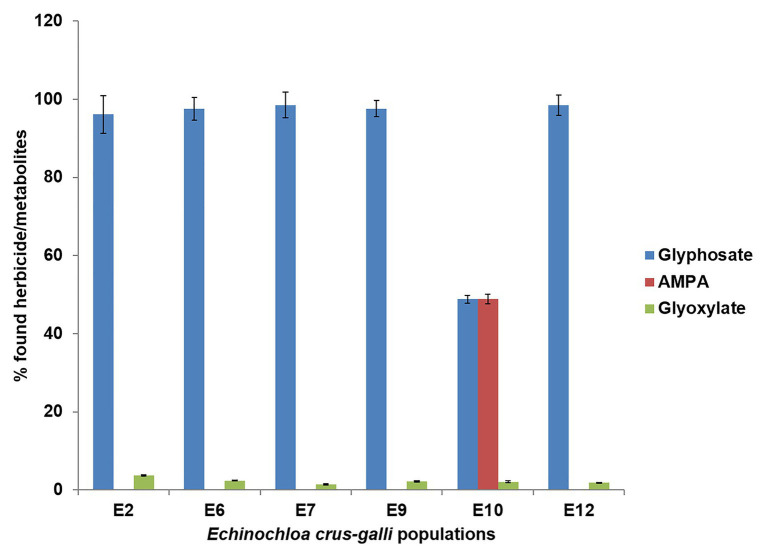
Glyphosate metabolism in glyphosate-resistant (R; E7 and E9), -very resistant (VR; E10 and E12), and -susceptible (S; E2 and E6) *Echinochloa crus-galli* plants 96 h after application at 360 g ae ha^−1^.

Metabolism of glyphosate was different between *E. crus-galli* populations at 96 HAT (Figure 5). Specifically, the accumulation of glyphosate in the E2, E6, E7, E9, and E12 populations was double that of the E10 population. The metabolism of glyphosate to AMPA and glyoxylate was 51%, while the rest of the populations studied remained unchanged and close to 90% (Figure 5). At least in part, metabolism had a crucial function in the response to glyphosate of the E10 population, from the VR group.

### Activity of the EPSPS

**Figure 6:**
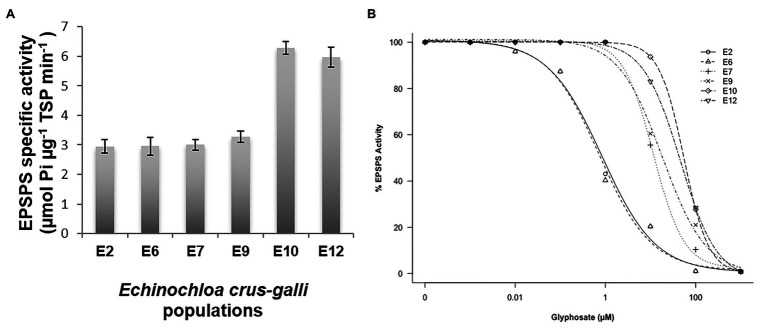
5-enolpyruvylshikimate-3-phosphate synthase activity in glyphosate-susceptible (S; E2 and E6), -resistant (R; E7 and E9), and -very resistant (VR; E10 and E12) *Echinochloa crus-galli* populations. **(A)** Mean of Basal EPSPS activity for glyphosate-susceptible and -resistant populations (*n* = 6). **(B)** EPSPS enzyme activity expressed as the percentage of the untreated control in leaf extracts of plants.

The basal activity of the EPSPS differed between the six *E. crus-galli* populations studied. The populations S (E2 and E6) and R (E7 and E9) had a similar EPSPS concentrations (2.95–3.0 μmol μg^−1^ TSP min^−1^), while the VR E10 and E12 populations had twice the target enzyme of glyphosate (6.0 μmol μg^−1^ TSP min^−1^) (Figure 6A). Inhibition of the EPSPS by glyphosate in plants from the S, R, and VR populations was achieved as herbicide concentrations increased. The R populations required between 16 and 25 times more herbicide to inhibit EPSPS by 50% in relation to the most susceptible population (E6, 0.7 μM glyphosate), while in the VR, such inhibition required between 46 and 55 μM herbicide (Table 3 and Figure 6B).

**Table 3:** Parameters of the equations and glyphosate concentrations (μM) required for a 50% reduction of 5-enolpyruvylshikimate-3-phosphate synthase (EPSPS) activity in different *Echinochloa crus-galli* populations.

Efficacy level	Population	d	b	R^2^	I_50_ (μM)	RF
Susceptible	E2	100.8	0.73	0.999	0.8	1.1
Susceptible	E6	100.9	0.75	0.999	0.7	--
Resistant	E7	100.7	1.30	0.999	11.7	15.8
Resistant	E9	101.2	0.90	0.999	18.1	24.5
Very Resistant	E10	100.0	1.58	0.999	54.8	74.0
Very Resistant	E12	100.3	1.107	0.999	45.6	61.6

RF, resistance factor (I_50_R/I_50_S).

## Discussion

Andalusia and Alentejo are the biggest regions in absolute terms of irrigated area with 1,295,918 ha, 29.35% of the total irrigated Spanish and Portuguese area. The dominant presence of localized irrigation stands out, which has been progressively increasing and represents 75% of the total main irrigation systems in these regions. The crops with the largest irrigated area are olive groves, citrus-trees, rice (flooding irrigation), wheat, and corn under direct sowing, as well as orchards and lately, for the last 20 years, new almond-tree plantations in an intensive regime. The use of glyphosate for many years under the row in perennial crops and also in fallow fields imposed massive selection pressure on the treated weeds, leading to the emergence of resistance, mainly in Mediterranean Europe (González-Torralva et al., 2012, 2014; Fernández-Moreno et al., 2017a,b; Amaro-Blanco et al., 2018; Vázquez-García et al., 2020a,b).

### Determining Resistance

*Echinochloa crus-galli*, a troublesome weed in rice, corn, and other perennial crops, is often controlled exclusively by chemical tools (Alarcón-Reverte et al., 2015; Nguyen et al., 2016; Fang et al., 2019; Vidotto et al., 2020). This work assessed the effect of repeated use of glyphosate in 13 populations of *E. crus-galli*. Using the accumulation rate of shikimic acid due to the EPSPS activity inhibition, it was observed that S populations significantly increased their shikimic level with respect to the putative resistant populations. This rapid screening allowed the separation of different levels of glyphosate susceptibility: S to glyphosate E2, E3, E4, and E6 and R- E1, E5, E7, E8, and E9, and VR- E10, E11, E12, and E13. The low values of GR_50_ and LD_50_, as those observed in S populations, are due to the fast and greater inhibition of the EPSPS, which results in a high accumulation of shikimate (Shaner et al., 2005). Inversely, low susceptibility to glyphosate and consequently little accumulation of shikimic acid, as observed in the R and VR *E. crus-galli* populations were consistent with the presence of one or more herbicide resistance mechanism, as found in different grass weed species (de Carvalho et al., 2012; Alarcón-Reverte et al., 2015; Vázquez-García et al., 2020b). This research also concluded that RF based in GR_50_ values separated these 13 populations in three groups, S, R, and VR (Figure 2). All resistant populations had values greater than 4, a requirement to be considered resistant (Heap, 2020; Vázquez-García et al., 2020a). In addition, the LD_50_ is widely employed to determine the herbicide rate need to kill the individuals of a weed population at 50%. Glyphosate label field dose recommended in Spain and Portugal is 1,080 g ae ha^−1^, which efficiently controlled the S populations E2, E3, E4, and E6, but not R populations E1, E5, E7, E8, and E9 or VR populations E10, E11, E12, and E13 of *E. crus-galli* (Table 2). This research revealed different levels of resistance to glyphosate in *E. crus-galli* collected in different crops of two large agricultural areas in Southern Spain and Central Portugal, where there is a variety of soils and climatic conditions. Weeds from different locations frequently show a differential response to herbicide, since each one has a unique genetic and ecological background, which is governed by climatic and edaphic conditions, type of crop where the weed developed, as well as cultural management crop tasks and the history of herbicide selection, among other agroecological factors (Shaner and Beckie, 2014; Jussaume and Ervin, 2016). In addition, it should also be considered that in each country, the glyphosate-based formulations, dose, time, and number of applications a year may vary, as well as the application technology used in each farm (Neve et al., 2014; Owen, 2016). Conversely, conditions of high temperature and relative humidity can contribute to improve the absorption and translocation of glyphosate, and effectiveness in monocots (Hatterman-Valenti et al., 2011; Nguyen et al., 2016; Fernández-Moreno et al., 2017a), which could help us understand the differences between *E. crus-galli* populations.

### Exploring the Mechanism Involved

The study of NTSR mechanisms was developed on two S-glyphosate (E2 and E6), two R- (E7 and E9), and two VR- (E10 and E12) populations. Epicuticular wax coating acts as an obstructive barrier against various herbicides. Some resistant and glyphosate-tolerant weeds have exhibited a non-uniform three-dimensional cover with a higher quantity of epicuticular waxes relative to their susceptible counterparts (Cruz-Hipolito et al., 2009, 2011). The E7, E9, E10, and E12 populations presented reduced absorption of ^14^C-glyphosate. However, this parameter is little studied and only in a few cases, such as Italian ryegrass (*Lolium multiflorum*), Johnsongrass (*Shorgum halepense*), and sourgrass (*Digitaria insularis*), has it been found to contribute to the lower susceptibility to glyphosate (Michitte et al., 2007; de Carvalho et al., 2012; Vila-Aiub et al., 2012). Differences in translocation occurred because the ^14^C glyphosate had moved nowhere once inside the leaf in R plants, whereas in S plants, glyphosate was uptake and translocated from the point of application to the rest of the shoots and roots in large quantities. Both absorption and impaired movement of glyphosate contributed to the resistance in the R and VR *E. cruss-galli* populations. It has been demonstrated in grass weeds that the main NTSR mechanisms involved in their resistance to glyphosate were those two (Vila-Aiub et al., 2012; Bracamonte et al., 2017; Gherekhloo et al., 2017).

Most plants do not have a high ability to metabolize glyphosate to non-toxic forms, favoring the death of plants. Some *Fabaceae* plants may be able to partially metabolize part of the absorbed glyphosate through glyphosate oxidoreductase (GOX), which cleaves the CN glyphosate bond forming amino methyl phosphonic acid (AMPA) and glyoxylate and, to a lesser extent, through a CP lyase, forming sarcosine and inorganic phosphate (Rojano-Delgado et al., 2010, 2012; Duke, 2011; Finley and Duke, 2020). Only four cases, among a wide range of studies on weeds resistant to glyphosate, reported metabolism as a resistance mechanism, showing evidence of glyphosate metabolites, such as AMPA or sarcosine (de Carvalho et al., 2012; González-Torralva et al., 2012; Pan et al., 2019). Among the six *E. crus-galli* populations studied, only the most resistant population E10 from the VR group was able to metabolize glyphosate (51%) to non-toxic metabolites (Figure 5). Aldo-keto reductase, a metabolic enzyme of plants, was found to be responsible for metabolizing glyphosate in glyphosate-resistant *Echinochloa colona* (Pan et al., 2019); however, molecular studies are necessary to establish or rule out the contribution of this enzyme in the glyphosate metabolism in the E10 *E. crus-galli* population.

Over the last two decades, research on the TSR mechanisms involved in glyphosate resistance have been carried out in a lot of monocot and dicotyledonous (Sammons and Gaines, 2014; Heap, 2020). Currently, two mechanisms within the target-site have been considered responsible for the resistance of weeds to glyphosate: (a) alteration/mutation at the encoding *EPSPS* gene that limit the interaction of glyphosate with the target enzyme and (b) overexpression/amplification of the target gene (Gaines et al., 2020). Differences between *E. crus-galli populations* in EPSPS enzyme activity were found with and without different glyphosate rates. Thus, R (E7 and E9) and VR (E10 and E12) populations had high I_50_ values (concentration of herbicide necessary to reduce EPSPS enzyme activity to 50%) with respect to the two glyphosate-susceptible E2 and E6 populations (Table 3 and Figure 6B). These results suggested that E7, E9, E10, and E12 populations were candidates that possess one or more effective mutation/s altering the coupling of the herbicide to the target enzyme (Salas et al., 2015; Fernández-Moreno et al., 2017a; Bracamonte et al., 2018; Morran et al., 2018). Additionally, the high glyphosate resistance values of VR populations E10 and E12 could be related to possible EPSPS overexpression, as suggested by a 2-fold increase in their EPSPS basal activity compared to E7 and E9 R populations. Differences in the EPSPS basal activity have already been documented in some grass weeds due to an overs-amplification of the *EPSPS* gene or even to an enhanced basal specific EPSPS activity in the absence of such amplification (Gaines et al., 2010; Alarcón-Reverte et al., 2015; Bracamonte et al., 2016). Further experiments are currently underway to unravel the TSR mechanisms present in these resistant *E. crus-galli* populations.

The close relative *E. colona* is also able to evolve different TSR and NTSR mechanisms to glyphosate, i.e., reduced translocation, point mutations, and enhanced metabolism. *Echinoclhopa colona* individuals with different and concerted TSR mechanisms were identified coexisting within different populations collected in the California Valley (Alarcón-Reverte et al., 2015). For example, some populations from Australia or United States exhibited mutations and others, reduced translocation (Nguyen et al., 2016; Nandula et al., 2018). Additionally, glyphosate metabolism has already been described in one *E. colona* population (Pan et al., 2019), which afterward also was shown to possess a Pro106Thr mutation (McElroy and Hall, 2020). Since each resistance mechanism usually confers different resistance levels, i.e., low to moderate resistance levels are associated with point mutations compared to other mechanisms (Sammons and Gaines, 2014), the evolution of one or more mechanisms within different populations should be associated mostly with differential selection pressures posed by glyphosate, among other factors. This seems to be the case for the *E. crus-galli* populations studied in this research. Two groups of populations were defined here according to the resistance levels: R and VR. Interestingly, previously, R populations survived 10–12 glyphosate applications at 720 or 1,080 g/ha, while VR ones, 12–15 applications almost always at 1,080 g/ha, according to historical herbicide records. Therefore, selection pressure was stronger with higher doses over more years in VR compared to R populations. Accordingly, reduced uptake and transport was detected in both groups, while metabolism was only detected in the most resistant VR population. Though TSR mechanisms were not investigated, EPSPS activity results suggested that mutations may be present in both R and VR populations, while overexpression might also be present in VR populations (E10 and E12), as pointed out by their ~2-fold increase in EPSPS basal activity. Future research is underway to underpin the TSR mechanisms that have evolved in these populations, which would confirm these hypotheses.

Combinations of multiple TSR and/or NTSR mechanisms in a single individual plant can also arise through outcrossing. Although *E. crus-galli* is a self-compatible and highly autogamous species, accidental cross-pollination can happen by wind (Maun and Barrett, 1986). The potential long-range pollen dispersal mediated by wind can facilitate the recombination of different resistance genes evolved either in different individuals of the same population or in distant populations of the species. Under the high selective pressure imposed by recurrent same-herbicide use, these rare recombinants, quickly fixed by predominant self-pollination, can be at immediate advantage, thus, spreading into the local population in a few generations (Bracamonte et al., 2017; Gaines et al., 2020).

In summary, the first record of resistance to glyphosate was confirmed in different populations of *E. crus-galli* harvested in contrasting croplands of the Iberian Peninsula. The resistance levels depended on diverse NTSR mechanisms, but it also involves putative TSR ones, which were differentially stacked by populations in response to the massive selection caused by glyphosate and other factors. These results concluded that resistance was independent of climate, type of crop, and geographic region, and that the glyphosate resistance level observed on the different populations of *E. crus-galli* studied increased by the intense use of the herbicide. The quick selection of multiple resistance mechanisms to glyphosate, TSR and NTSR, including enhanced metabolism, is very worrying. Farmers must implement strategies of weed control, including available cultural and non-chemical strategies, as well as other herbicides with different modes of action to glyphosate in integrated weed management programs, to alleviate the herbicide selection pressure and suppress/reduce the evolution resistance.

## Data Availability Statement

The original contributions presented in the study are included in the article/supplementary material, and further inquiries can be directed to the corresponding author.

## Author Contributions

JGV-G and RDP: general idea and designed the experiments. JGV-G, JP, and RDP collected the different populations. JGV-G, AMR-D, JT, ID, JP, and RDP performed the research. RA-dlC, JT, ID, JP, and RDP analyzed and validated the results. All authors contributed to the article and approved the submitted version.

### Conflict of Interest

The authors declare that the research was conducted in the absence of any commercial or financial relationships that could be construed as a potential conflict of interest.
